# Impact of Multi-Factor Features on Protein Secondary Structure Prediction

**DOI:** 10.3390/biom14091155

**Published:** 2024-09-13

**Authors:** Benzhi Dong, Zheng Liu, Dali Xu, Chang Hou, Na Niu, Guohua Wang

**Affiliations:** College of Computer and Control Engineering, Northeast Forestry University, Harbin 150040, China; nefudbz@nefu.edu.cn (B.D.); zhengliu@nefu.edu.cn (Z.L.); nefuxdl@nefu.edu.cn (D.X.); houchang@nefu.edu.cn (C.H.)

**Keywords:** protein, secondary structure prediction, multi-factor

## Abstract

Protein secondary structure prediction (PSSP) plays a crucial role in resolving protein functions and properties. Significant progress has been made in this field in recent years, and the use of a variety of protein-related features, including amino acid sequences, position-specific score matrices (PSSM), amino acid properties, and secondary structure trend factors, to improve prediction accuracy is an important technical route for it. However, a comprehensive evaluation of the impact of these factor features in secondary structure prediction is lacking in the current work. This study quantitatively analyzes the impact of several major factors on secondary structure prediction models using a more explanatory four-class machine learning approach. The applicability of each factor in the different types of methods, the extent to which the different methods work on each factor, and the evaluation of the effect of multi-factor combinations are explored in detail. Through experiments and analyses, it was found that PSSM performs best in methods with strong high-dimensional features and complex feature extraction capabilities, while amino acid sequences, although performing poorly overall, perform relatively well in methods with strong linear processing capabilities. Also, the combination of amino acid properties and trend factors significantly improved the prediction performance. This study provides empirical evidence for future researchers to optimize multi-factor feature combinations and apply them to protein secondary structure prediction models, which is beneficial in further optimizing the use of these factors to enhance the performance of protein secondary structure prediction models.

## 1. Introduction

The protein secondary structure is determined by the interactions of hydrogen bond donor and acceptor residues on the polypeptide backbone and is a localized three-dimensional structural fragment formed prior to the folding of proteins into a tertiary structure, bridging the gap between the primary and tertiary structures [1]. As an important component of proteins, the protein secondary structure lays the foundation for the formation of the tertiary structure, which is important in understanding the function and stability of proteins [2]. In recent years, the use of computational methods for secondary structure prediction has received increasing attention as a low-cost and efficient alternative to wet experiments [3]. In practical applications, protein secondary structure prediction methods are required to be not only superior in performance, but also to have a high degree of consistency and scientific validity in data representation and process interpretability. By effectively utilizing protein-related features for secondary structure prediction, the prediction process can be significantly improved, and the accuracy and efficiency of the prediction can be enhanced [4].

In the previous studies, scientists explored various methods to improve protein secondary structure prediction techniques, and most of the studies mainly focused on different input data features. For example, early PSSP methods were mainly based on protein sequence information [5]. The PHD proposed by Burkhard Rost et al. [6] automatically processed amino acid sequences through a contour alignment algorithm and used feed-forward neural networks to correlate the sequences with the structures to perform secondary structure prediction. Similarly, Wang et al. [7] introduced an advanced convolutional neural network combined with a self-monitoring conditional random field, starting from sequences. This approach is adept at capturing intricate sequence–structure relationships and accounting for the interdependencies between structures. With the progress of the research, position-specific scoring matrices (PSSM) [8] are also being gradually introduced into PSSP. In 2020, Cheng et al. [9] proposed a PSSP method combining CNN and LSTM, which makes full use of PSSM matrices through probabilistic weighting and cross-validation to enhance the prediction accuracy. PSSM is gradually becoming an essential factor in secondary structure prediction along with protein sequences [10,11,12]. To enhance prediction accuracy and data richness, the physicochemical properties of amino acids have been increasingly incorporated. Jacek Błazewicz et al. [13] examined the significance of various amino acid properties in predicting protein secondary structures to address the challenge of forecasting the secondary structure of related proteins. Li et al. [14] introduced a hybrid coding prediction method that merges multiple physical and chemical properties of amino acids with secondary structures to create a multidimensional fusion coding, utilizing support vector machines for prediction. As the research progresses, a growing number of scientists are concentrating on exploring and optimizing input features to boost prediction accuracy and efficiency.

Although these studies have markedly improved the performance of protein secondary structure prediction, there are still few studies on the properties of these factors and their importance in the prediction task, and there is a lack of quantitative analyses of the role these multi-factor features play in secondary structure prediction. We evaluated four mainstream factors, including the physical and chemical properties of amino acids, trend factors, position-specific scoring matrix, and amino acid sequence, and we analyzed their importance in the performance of PSSP and gave explanations for their impact on the prediction process, enabling them to be better matched with computational models in applications to achieve reduced computational overhead and improved prediction accuracy.

Secondary structure trend factors (also known as amino acid compositional tendencies) in PSSP reflect the intrinsic properties of amino acids and the compositional differences across structures with significantly different effects on secondary structure [15]. For example, in the α-helical structure, certain amino acids, such as alanine and glutamic acid, have a high preference for side chains that are able to form hydrogen bonds and internal electrostatic interactions, thus positively affecting the stability of the helical structure. Glycine and alanine are more common in β-folded structures as their side chains do not interfere with β-sheet formation [16]. It was found that there is a significant correlation between the prediction of secondary structure and the chemical and physical characteristics of amino acids [17]. The prediction accuracy of hydrophobic residues is usually higher, while that of hydrophilic residues is lower [18]. In addition, observations showed that in the three-state secondary structure, there were differences in the amino acid predictions in the middle and terminal regions: the prediction errors in the middle region were mainly concentrated on a few residues, whereas those in the terminal region were more significant. Regarding the basic factors incorporated in this study—the protein amino acid sequence and PSSM—they have been widely regarded in the field of PSSP as underlying factors.

The main contributions of this paper are as follows:

- Firstly, we conducted a comprehensive analytical study of the multi-factor features and evaluated the effect of multi-factor combinations and the independent action features of each factor using machine learning methods. Through this analysis, we can not only explore the interactions among the factors, but also understand the independent features of each factor in detail, so as to verify and understand the role of multi-factor features in protein secondary structure prediction more comprehensively.
- Secondly, we quantitatively assessed the prediction performance of protein three- and eight-state secondary structures by combining multi-factor features. By using classical and explanatory machine learning models, we not only reveal the synergistic effects of multiple factors, but also analyze their specific contributions in different prediction tasks.

For the above work, we have not only analyzed the combinatorial and independent action properties of multiple factors through a series of experiments, but also highlighted the impact of various factors on predicting protein secondary structure. These research results not only deepen our understanding of the mechanism of the role of each factor in the prediction task, but also provide an important reference for improving the prediction performance in the future.

## 2. Materials and Methods

### 2.1. Datasets

In this study, we used the standardized dataset SCRATCH-1D [19], the openly accessible dataset CB513 [20], and CASP11 and CASP10 (https://predictioncenter.org/) accessed on 19 June 2024. The SCRATCH-1D dataset contains secondary structure information for 8059 proteins derived from X-ray crystallographic structure resolution at no less than 2.5 angstroms and without discontinuities. To ensure the reliability and fairness of the results, we limited the sequence similarity in the dataset to less than 25%. We used the SCRATCH-1D dataset for model training and the CB513, CASP11, and CASP10 datasets as independent test sets to evaluate the generalization ability of the models. To ensure the accuracy of the out-of-sample evaluations, we pre-processed the CB513 dataset by removing entries containing unnatural residues (e.g., residues containing ‘X’) and finally retained 471 protein samples. Similar processing was performed on the CASP11 and CASP10 datasets. This training–testing split approach allowed us to report on out-of-sample metrics and validate the model performance on unseen data, ensuring the applicability and comparability of our findings.

In addition, we used the PSI-BLAST [21] tool for protein sequence comparison. The comparison process was performed with several iterations, and the threshold was set to 0.001 to generate a PSSM matrix reflecting the score of each amino acid at various positions, showing its importance in the sequence. In order to assess the influence of amino acid chemical properties on the secondary structure prediction, the property parameters needed for the study were selected by looking at the AAindex database [22], including the acid–base properties of the acidic and basic protons (pKa1 and pKb2), hydrophobicity (H), and isoelectric point PH (pl). We indirectly considered the effect of the protonation state on the secondary structure of the protein by using the pKb2, pI, and pKa1 values of the amino acids, which together reflect the charge changes in the amino acids and their potential impact on the predictions. We selected them according to their effects on different elements of the secondary structure; the details of these effects are given in Table 1.

In calculating the secondary structure trend factor, we assumed a certain regularity in the distribution of the secondary structures. By analyzing the datasets, the frequency of occurrence of each secondary structure was determined; this allowed us to quantify its relative importance. The trend factors calculated in this way helped us to assess the performance of the PSSP methods more comprehensively.

### 2.2. Characterization of Multi-Factor Features

In this study, we used multi-factor features containing four different factors with the different treatments of these data. Firstly, we applied one-hot coding to the protein amino acid sequence data. This approach converts each amino acid residue into a unique binary vector, where each amino acid occupies a distinct position in the vector. This encoding not only provides clear positional information for each residue, but also lays the foundation for subsequent data fusion and processing, allowing us to understand the secondary structure details of the proteins in a more intuitive manner.

To further enhance the input features, we utilized the PSI-BLAST tool to compare protein sequences and generated a PSSM matrix of *n* × 20, where n denotes the protein sequence’s length and 20 amino acid types. Each value in the PSSM reflects the specificity scores of the amino acids at a particular position, and these scores show the relative importance and evolutionary conservation. This information is essential for capturing key structural features in protein sequences.

In order to select appropriate parameters of the amino acid properties, we considered their different effects on the secondary structure of proteins. Hydrophobic amino acids influence the structure formation of proteins by binding to water molecules, whereas polar and nonpolar amino acids play different roles in their interaction with proteins, thus affecting their stability. Acid–base amino acids influence the structure and stability of proteins by regulating the charge distribution within them. We selected four key amino acid property parameters, pKa1, pKb2, pI, and H, with the aim of exploring their impact on PSSP.

**Figure 1:**
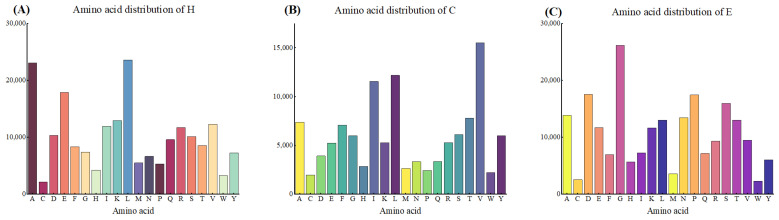
Schematic representation of secondary structure distribution: (**A**) Frequency distribution of secondary structure H in the dataset; (**B**) frequency distribution of secondary structure E in the dataset; (**C**) frequency distribution of secondary structure C in the dataset.

Secondary structure trend factors were calculated to identify and quantify the secondary structure patterns (e.g., α-helices, β-folds, and irregular coils, denoted by H, E, and C, respectively) in protein sequences, and a schematic distribution of the number of secondary structures is given in Figure 1. We identified these trend factors by analyzing the prevalence of secondary structures in the datasets. The introduction of these factors helps to reveal the distribution pattern of the protein structures, thereby enhancing the predictive accuracy.

It was found that there are certain mathematical patterns in the secondary structure distribution of proteins in different datasets, as evidenced by the significant variability in the frequency of occurrence of specific amino acids in different secondary structure conformations (H: α-helical, E: β-folded, C: randomly curled). We quantified the distribution tendency of each amino acid in these secondary structure conformations by statistical analysis. For example, certain amino acids (e.g., alanine and glutamic acid) occur significantly more frequently in α-helices than others, whereas glycine and alanine are more common in β-folded structures. Based on these statistical results, we calculated the trend factor of amino acids in different secondary structures to reflect their preference in specific structures. This trend factor is calculated as shown in Equation (1) and is used to quantify the relative preference of amino acids in each secondary structure.

(1) $$Pi=\frac{Ai}{Ti},\;i\in \left\{H,E,C\right\}$$

wherein the score for each amino acid may be expressed as Ai, where Ai is the tendency score for that amino acid in a particular secondary structure conformation (e.g., *H*, *E*, or *C*), reflecting a preference for that amino acid in that secondary structure; Ti is the total score for that secondary structure conformation; and Pi is the tendency factor score for the secondary structure.

Subsequently, the frequency information is converted into normalized experimental data by a normalization operation, the principle of which is shown in Equation (2):

(2) $$Pi=\frac{Pi-Pmin}{Pmax-Pmin}$$

**Figure 2:**
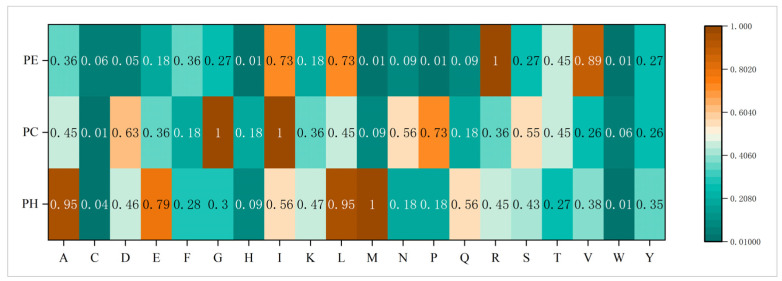
Secondary structure propensity scores.

These steps allow us to capture and compare secondary structure trends in different protein sequences more efficiently. Figure 2 illustrates the specifics of the secondary structure trend factor scores in this study.

Multi-factor features are useful because they combine the sequence information, position specificity score, and amino acid properties and trend factors to provide a comprehensive characterization. This comprehensive feature representation can better capture the intricacies of protein secondary structure and enhance the predictive model’s efficacy. By integrating multiple features, more comprehensive and accurate data support can be provided for secondary structure prediction.

### 2.3. Machine Learning Methods Used in Factor Evaluation

To analyze the effectiveness of the multi-factor approach suggested in this study for PSSP, we used four representative and classical machine learning methods for comparative studies, including the support vector machine (SVM) [23], random forest (RF) [24], K-nearest neighbor [25], and naive Bayes [26]. With this multi-factor approach, we aim to demonstrate its significant advantages and practical applications in protein secondary structure prediction.

#### 2.3.1. Support Vector Machine

Based on statistical learning theory and the principles of minimizing structural risk, the support vector machine (SVM) is a representative machine learning algorithm. Compared with other machine learning algorithms, SVM performs better in many cases because it is able to effectively control the capacity of the classifier, thus reducing the risk of overfitting.

In essence, SVM aims to identify the optimal hyperplane by utilizing support vectors, as illustrated in Equations (3)–(5). In this case, we obtain Equation (3) by finding the sum of the maximum distance from two dissimilar support vectors to the hyperplane:

(3) $$d_{max}=\frac{2}{\left||\omega |\right|}$$

Such dissimilar points are called “support vectors”. In order to find the optimal hyperplane, i.e., the hyperplane with the maximum spacing distance, the problem is transformed into one requiring the solving of an extrema. To solve this problem, the objective function of the dyadic problem is obtained by introducing the Lagrange multiplier method, as in Equation (4):

(4) $$L\left(\omega ,b,\alpha \right)=\frac{{\left|\left|\omega \right|\right|}^{2}}{2}-\sum_{i=1}^{n}\alpha_{i}(y_{i}\left(\omega^{T}x_{i}+b\right)-1)$$

In this equation, the expressions for ω and b are obtained by making the partial derivatives of L (ω, b, α) with respect to ω and b zero. This in turn transforms the problem into a problem requiring the solving the extremum of the Lagrange multiplier α. Subsequently, we employ the sequential minimal optimization (SMO) algorithm, and the (Karush–Kuhn–Tucker) condition is satisfied to obtain the final hyperplane model expression, as in Equation (5).

(5) $$f\left(x\right)=\omega^{T}x_{i}+b=\sum_{i=1}^{n}\alpha_{i}y_{i}x_{i}^{T}x+b$$

Support vector machines (SVMs) attempt to find the optimal hyperplane to classify data by utilizing support vectors in a high-dimensional space. The goal of the method is to efficiently process multi-factor features and capture complex patterns in the data. This property of SVMs is also used in many deep learning methods, such as recurrent neural networks (RNN), which identify complex structures in data through high-dimensional feature representations. The application of SVMs to high-dimensional data processing provides further optimization and improvement of other machine learning and deep learning methods and the foundation.

#### 2.3.2. Random Forest

The random forest (RF) model is a commonly employed supervised learning technique, extensively utilized for classification tasks, including PSSP. Its main advantage is its ability to reduce the risk of overfitting by integrating multiple decision trees to improve the overall model performance. In this study, to solve the multi-factor problem, we introduced random forests. The random forest model adopts the bagging strategy, as shown in Equation (6), i.e., it randomly constructs multiple decision trees and integrates them together; it is suitable for dealing with nonlinear problems and a large number of sample features and is highly compatible with the features of multi-factor data in this study.

(6) $$f\left(x\right)=\frac{1}{M}\sum_{m=1}^{M}f_{m}(x)$$

In this process, the base learners are non-dependent and independent of each other, and parallel training can be achieved, in order to effectively manage diverse data and enhance both the prediction accuracy and the model’s ability to generalize.

Random forests (RFs) improve model robustness and generalizations by integrating multiple decision trees. The method is suitable for handling multi-factor data and can perform well in high-dimensional and nonlinear problems. Similar integration strategies have been applied in deep learning, such as model ensembling and multi-task learning, which improves the overall model performance by integrating different models or tasks. These features of random forests provide valuable ideas for dealing with complex data.

#### 2.3.3. K-Nearest Neighbor

K-nearest neighbor (K-NN) is an intuitive and straightforward instance-based learning algorithm within supervised learning. In the task of PSSP, the algorithm performs well mainly because of its ability to handle multi-factor data and high-dimensional feature space effectively. The K-NN algorithm uses the information of local neighborhoods to make predictions, which makes the algorithm excel in this task, given the strong correlation between local sequences of proteins and secondary structures. In the classification problem, K-NN uses majority voting rules. When K = 1, the amino acid in each protein sequence is assigned to the secondary structure corresponding to its nearest neighbor, i.e., one secondary structure is predicted for each amino acid. In the testing phase, K-NN identifies the k training samples in the training set that is closest to the test samples based on the feature similarity using a distance metric (e.g., Euclidean distance) and performs classification based on the information of these neighbors. Euclidean distance is used to measure the similarity between amino acids, which is computed as depicted in Equation (7).

(7) $$d\left(x,y\right)=\sqrt{{\left(x_{1}-y_{1}\right)}^{2}+{\left(x_{2}-y_{2}\right)}^{2}}$$

The K-nearest neighbor (K-NN) algorithm classifies samples through a voting mechanism and a distance metric mechanism, and it is particularly suited to handling high-dimensional and multi-factor data. K-NN relies on the information of local neighborhoods for prediction, and this approach has similar applications in some deep learning techniques, such as the attention mechanism, which selects important features by calculating the correlation weights between the input features to select important features. These mechanisms are effective in focusing on key features when dealing with complex biological data and help improve the prediction performance of the model.

#### 2.3.4. Naive Bayes

Naive Bayes (NB) is a statistical method whose key assumption is the conditional independence between individual features; it belongs to the generative model of supervised learning with a solid mathematical theoretical foundation. Due to its simplicity, efficiency and good interpretability, naive Bayes has become one of the preferred methods for many classification tasks. In the task of protein secondary structure prediction, the naive Bayes algorithm, with its probabilistic model property, shows certain advantages in dealing with high-dimensional multi-factor data. The objective of this study is to utilize the naive Bayes algorithm for PSSP. Although the naive Bayes algorithm is based on the conditional independence assumption, which may be limited when dealing with complex correlated data, it can still provide competitive performance and results in the context of multi-factor high-dimensional data. The basic principle of naive Bayes as a probabilistic classification method is shown in Equation (8).

(8) $$P=(C=c|X_{1}=x_{1},\ldots .X_{n}=x_{n})$$

The model estimates the conditional probability of each variable $X_{k}$ given the class label C using the training data and then applies it to the Bayes rule to calculate the probability of a specific instance of C given $X_{1}$ up to $X_{n}$. The post-validation probability of the class variable (expressed by the naive Bayes classifier) is thus shown in Equation (9):

(9) $$h_{nb}\left(x\right)=arg\;\underset{c\in y}{\max}\;P(c)\prod_{i=1}^{d}P(x_{i}|c)$$

where P(c) is the prior probability of the category, and $P(x_{i}|c)$ is the conditional probability of feature $x_{i}$ of the category under condition c. By learning from the training data, these probabilities can be effectively estimated to achieve accurate classification of new samples.

The naive Bayes (NB) algorithm is based on the assumption of the conditional independence of features and classifies by calculating posterior probabilities. This approach is known for its computational simplicity and efficiency and is particularly suitable for processing high-dimensional multi-factor data. The idea of naive Bayes is also reflected in certain deep learning methods, such as in convolutional neural networks, where large-scale data can be processed by simplifying the computation. These features of naive Bayes inform the design of more efficient models.

### 2.4. Experimental Environment and Evaluation Parameters

#### 2.4.1. Experimental Environment

The following hardware devices were used in this study:

- CPU: Intel Xeon Gold 5218R, 2.10 GHz;
- GPU: RTX 2080Ti (11 GB), cuda11.1;
- Memory: 64 GB.

The setup included Ubuntu 16.04 as the operating system and Python 3.7 for programming, and the PyTorch framework was utilized to develop the model and perform the experiments.

#### 2.4.2. Performance Evaluation

In order to assess the influence of multi-factor features on performance in protein secondary structure prediction and the effect of the factors, we used the precision ($Q_{m}$) as one of the evaluation indexes. By comprehensively calculating the parameters of each amino acid, we were able to assess the specific contribution of these factors, as detailed in Equation (10):

(10) $$Q_{m}=\frac{\sum_{i=1}^{m}A_{i}}{N}$$

where $Q_{m}$ represents the precision; the value of m corresponds to the number of classes of three-state secondary structures; N represents the total number of amino acid residues; and $A_{i}$ denotes the count of accurately predicted amino acids.

Segment overlap (Sov) is an important metric used to assess the accuracy of protein secondary structure prediction [27]. It is mainly used to measure the degree of similarity between the predicted secondary structure and the actual secondary structure, and its calculation formula is as follows (see Equations (11) and (12)):

(11) $$Sov=\frac{100*\sum_{S0}\left[\frac{minov\left(S_{1},S_{2}\right)+\sigma \left(S_{1},S_{2}\right)}{maxov\left(S_{1},S_{2}\right)}\cdot length\left(S_{1}\right)\right]}{N_{Sov}}$$

(12) $$\sigma \left(S_{1},S_{2}\right)=min\left\{\begin{array}{c} \;\;\;\;\;maxov\left(S_{1},S_{2}\right)-minov\left(S_{1},S_{2}\right)\\ minov\left(S_{1},S_{2}\right)\\ int\left[len\left(S_{1}\right)\right]/2\\ int\left[len\left(S_{2}\right)\right]/2 \end{array}\right.$$

where $S_{1}$ and $S_{2}$ denote the predicted and actual secondary structure fragments, respectively; $minov\left(S_{1},S_{2}\right)$ and $maxov\left(S_{1},S_{2}\right)$ denote the minimum and maximum overlap lengths between $S_{1}$ and $S_{2}$, respectively; $len\left(S_{1}\right)$ is the length of fragment $S_{1}$; $N_{Sov}$ is the total number of secondary structure fragments used to calculate Sov; and $\sigma \left(S_{1},S_{2}\right)$ is the compensation term used to adjust the overlap by selecting the minimum of multiple values.

## 3. Experiments and Results

### 3.1. Experimental Hyperparameter Optimization and Principles of Method Action

#### 3.1.1. Experimental Hyperparameter Optimization Exploration

Prior to the overall experiment, we first performed hyperparameter exploration of the model to find the best hyperparameter settings. This process aimed at optimizing the performance of the model to more accurately verify the effect of multi-factor features on protein secondary structure prediction. By carefully adjusting the hyperparameters, we were able to improve the predictive ability of the model and ensure the reliability and validity of the experimental results, the details of which can be found in Table 2.

Support Vector Machines (SVMs): There are significant differences in the ability of different SVM kernels to model data relationships. The linear kernel is suitable for linearly differentiable data but has limited ability to handle complex nonlinear relationships and therefore performs poorly, while the RBF kernel performs the best because it is highly adaptable and can handle complex nonlinear relationships effectively. The polynomial kernel function captures some nonlinear relationships but does not perform as well as the RBF with complex data and high-dimensional problems. The sigmoid kernel function is usually the least effective because it is weakly adaptable to complex data and is prone to overfitting or underfitting.

Random Forest (RF): The performance of random forests usually improves with an increase in the number of decision trees, as more trees better capture the complex patterns of the data. However, when the number of trees is too high, the model may introduce noise or overfitting, which can affect the overall performance. In our experiments, a setting of 120 decision trees performed best, effectively balancing complexity and preventing overfitting.

K-Nearest Neighbor (KNN): In KNN, smaller K values (e.g., K = 1) tend to lead to overfitting because the model is too sensitive to training data noise. A moderate K value (e.g., K = 3) can better balance the data noise with the real model and performs best. Larger K values (e.g., K = 4) can lead to underfitting because the model may not be able to effectively capture the detail and complexity in the data.

Naive Bayes: Because naive Bayes models assume that features are independent of each other, they have a simple structure and low hyperparameter dependence. This allows naive Bayes models to be used in practical applications for fast and efficient modeling and prediction without complex parameter tuning.

#### 3.1.2. Experimental Setup and Methodology Rationale

In order to evaluate the four factor features more rationally, we set the following parameters for the four selected machine learning algorithms to run:

(1) Support Vector Machine (SVM): The radial basis function (RBF) is used as the kernel function. SVM maps the protein sequence data into a feature space with high dimensionality and constructs classifiers through support vectors to improve the prediction accuracy.

(2) Random Forest (RF): The number of decision trees is set to 120, and the maximum depth of each decision tree is set to 15. By selecting a subset of attributes on each decision tree node, random forests select the optimal attributes by voting to identify the protein secondary structure efficiently and to reduce the generalization error.

(3) K-Nearest Neighbor (K-NN): The value of K is set to 3. K-NN computes and classifies test samples as they are entered, using local neighborhood information to improve prediction accuracy and generalization.

(4) Naïve Bayes (NB): The “assumption of conditional independence of attributes” is used to make predictions by calculating the likelihood of each amino acid feature with respect to the corresponding secondary structure category.

### 3.2. Performance Comparison of Multi-Factor Feature Combinations for Three-State Secondary Structure Prediction

**Figure 3:**
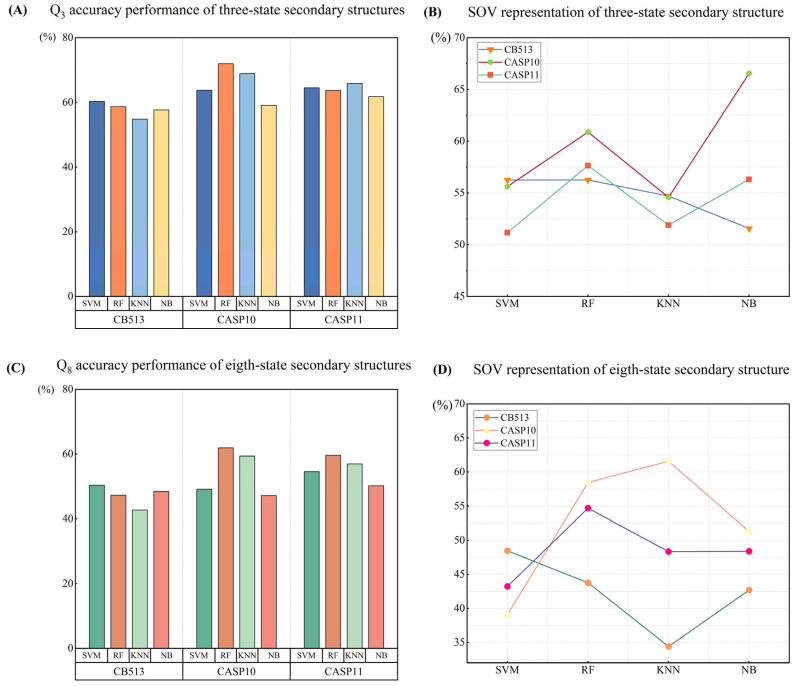
Performance plots for three-state and eight-state secondary structure predictions are shown. (**A**) Illustration of Q3 accuracy for three-state secondary structure; (**B**) illustration of SOV performance for three-state secondary structure; (**C**) illustration of Q3 accuracy for eight-state secondary structure; (**D**) illustration of SOV performance for eight-state secondary structure.

To gain a thorough understanding of the contribution of each multi-factor feature in secondary structure prediction and the impact of the various methods on the process, we evaluated the performance of these models using identical multi-factor input data. Table 3 demonstrates the efficacy of the four machine learning methods in the task of protein secondary structure prediction, where we use $Q_{3}$ accuracy and fragment overlap (Sov) as evaluation metrics. In the table, the prediction accuracy and the fragment overlap of each model under different test datasets (CB513, CASP10, CASP11) are listed, and they are visualized in Figure 3A,B.

From Table 3 and Figure 3A,B, it can be seen that different machine learning models show different performances on different datasets. SVM shows high $Q_{3}$ accuracy on all the datasets, especially on the CASP10 dataset, which reaches 63.73%. RF performs particularly well on the CASP10 dataset, with a $Q_{3}$ accuracy of 71.92%, and Sov also reaches 60.88%, showing a strong comprehensive prediction ability. K-NN overall good performance on the CB513 dataset but achieves a $Q_{3}$ accuracy of 65.85% on the CASP11 dataset. Naive Bayes has the best Sov performance on the CASP10 dataset at 66.55%, but its performance on the other datasets is relatively weak.

Overall, these results further validate the key role of multi-factor features in protein secondary structure prediction. The importance and validity of multi-factor features are demonstrated by the fact that the accuracy and reliability of the prediction can be significantly improved by the combined use of amino acid sequence information, PSSM matrix, amino acid properties, and trend factors.

### 3.3. Performance Comparison of Multi-Factor Feature Combinations for Eight-State Secondary Structure Prediction

In this research, we not only explored the three-state protein secondary structure prediction, but also further extended it to the more complex eight-state protein secondary structure prediction. This study employs the same dataset and methodology and aims to comprehensively evaluate the performance of our proposed multi-factor features in different prediction tasks. By comparing the performance of different machine learning models in eight-state secondary structure prediction, we hope to further validate the importance of multi-factor features in improving prediction accuracy and reliability.

With the experimental results in Table 4 and Figure 3C,D, we can see that multi-factor features play a significant role in different machine learning models and different prediction tasks. This not only verifies the criticality and universality of multi-factor features in protein secondary structure prediction, but also provides an important reference for future research in this field. These findings lay a solid foundation for the further enhancement of the accuracy of protein secondary structure prediction and demonstrate the great potential of multi-factor features in this field.

### 3.4. Importance Analysis of Multi-Factor Features

In this section, we examine the significance of various factors from distinct perspectives, respectively. First, from a methodological perspective, the synergistic properties of multiple factors and their applicability conditions are explored to reveal how different machine learning methods can exploit the synergistic effects of these factors to improve the prediction performance. Second, from the factor perspective, we split the multiple factors and conduct an in-depth study of each factor, aiming to explore the independent action properties of each factor. Through these two parts of the study, we seek to comprehensively understand the mechanism of multiple factors in protein secondary structure prediction.

#### 3.4.1. Importance Analysis of Different Features under the Same Methods

**Figure 4:**
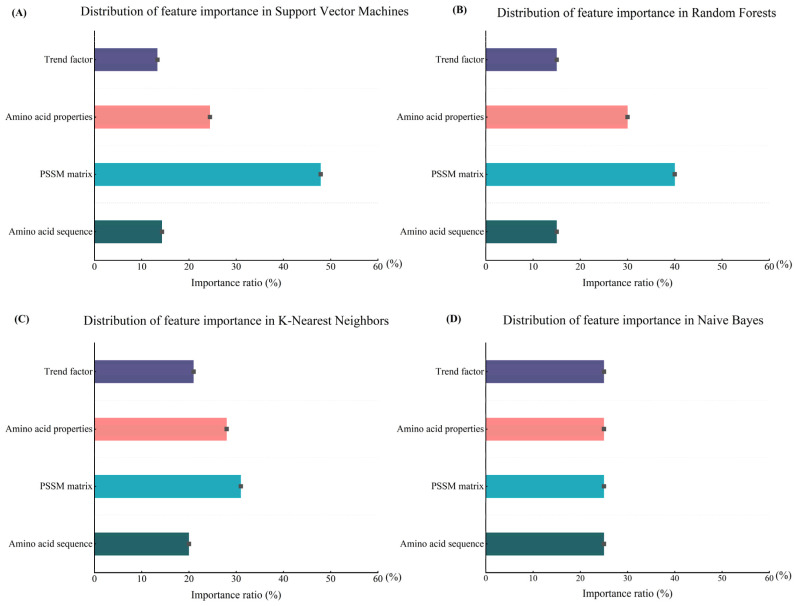
Schematic representation of the importance analysis of different features across various machine learning methods. (**A**) Visualization of feature significance for SVM; (**B**) visualization of feature significance for RF; (**C**) visualization of feature significance for K-NN; (**D**) visualization of feature significance for NB.

To achieve a deeper insight into the overall action properties of the four factors, we conducted an experiment on the importance analysis under different methods. This experiment aims to explain the synergistic effect of multiple factors and reveal the principle of their synergistic effects by analyzing the performance of different machine learning methods. Through this method, we can analyze the role features of each factor more deeply. In Figure 4, we show the results of the importance analysis of multiple factor features using four machine learning methods. The results offer strong support for understanding the performance and the underlying mechanisms of each factor in different algorithms.

As a result of the study, we found that the importance of these multiple factor features varies significantly in different machine learning methods. In SVM, the results show that the PSSM matrix has a significant influence on SVM. This can be attributed to the high-dimensional feature mapping capability of SVM, where the PSSM matrix can provide rich positional and evolutionary information, thus enabling SVM to better capture the complex patterns implicit in protein sequences. Amino acid properties and trend factors also contribute to the prediction accuracy to some extent, but their importance is relatively low.

In random forests, the generalization ability of the model is improved by constructing multiple decision trees and integrating their results. In this approach, the importance of the amino acid properties was significantly increased, probably because RF was able to more fully exploit the diversity and complexity of amino acid properties when choosing the delineation attributes. The PSSM matrix was still the most important factor, which demonstrates its key role in protein secondary structure prediction.

In K-nearest neighbor, the numerical characteristics of the factors have a greater impact on the final results as K-NN relies on a distance metric for classification. The higher importance of the PSSM matrix and amino acid properties in K-NN suggests that the detailed feature information provided by these factors contributes to a more accurate calculation of distances and classification. The amino acid sequence and trend factors also contributed significantly, reflecting the sensitivity of K-NN to local neighborhood information.

In naive Bayes, the importance of all four factors is about 25%. It is important to note that in naive Bayes, due to its mathematical theoretical nature, all features are assumed to be conditionally independent, which means that each feature independently affects the classification result for a given category. Therefore, theoretically, there will be no one feature that is significantly better than the others, and each feature is considered equally important, which is one of the reasons why naive Bayes is easy to interpret. Nonetheless, by utilizing the independent information from each factor, naive Bayes is still able to provide valid predictive results.

From the above analysis, it can be seen that there are significant differences in the way the factors are utilized by different machine learning methods. SVM and RF perform well in dealing with complex features, especially with the high importance of the PSSM matrix, which suggests that they are able to effectively utilize the location information and the evolutionary information to improve the prediction accuracy. In contrast, naive Bayes is not able to adequately capture the interactions between factors due to its attribute of conditional independence assumption; thus, the importance of each factor is equal. K-NN, on the other hand, excels in the exploitation of the amino acid properties and the trend factor, which shows its sensitivity to local neighborhood information. These differences reflect the respective strengths and limitations of the different algorithms when dealing with the task of protein secondary structure prediction.

#### 3.4.2. Importance Analysis of Each Feature under Different Methods

**Figure 5:**
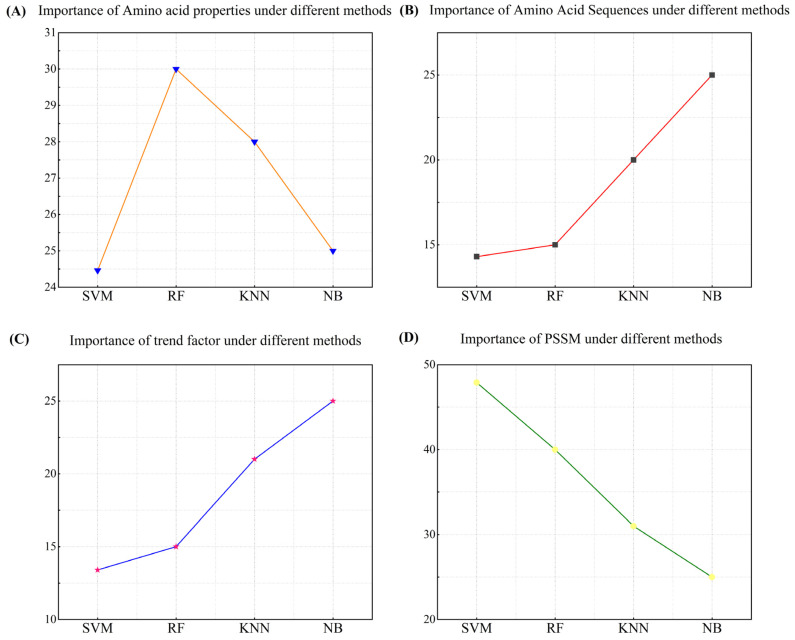
Importance analysis of each factor under different methods. (**A**) Importance analysis of amino acid properties; (**B**) importance analysis of amino acid sequences; (**C**) importance analysis of trend factors; (**D**) importance analysis of PSSM matrix.

In order to gain insight into the independent action features of each of the four factors, an importance analysis was conducted for each factor individually. This method explains the features of the action of each factor by studying each factor individually, aiming to find the most suitable conditions for the action of each factor. In this subsection, we only analyze and explain the features of the factors, and their principles of action in the method are not repeated, as details can be found in the previous subsection. We present the results of this research in Figure 5.

The importance distribution of the amino acid properties under different the machine learning methods is shown in Figure 5A. Amino acid properties reflect the physicochemical properties of each amino acid, such as hydrophobicity and acid–base properties. It is shown that there is a correlation between the prediction of secondary structure and the physicochemical properties of the amino acids. The prediction accuracy of hydrophobic residues is usually high, while the prediction accuracy of hydrophilic residues is relatively low. The β-folded residues were poorly represented in the dataset, which affected the accuracy of the prediction results to some extent. In contrast, the residues in the α-helix and coiled-coil structures were more evenly distributed, which may lead to better secondary structure prediction results. Therefore, information on the nature of amino acids can help the model to identify the relationship between the structure and amino acids, which has a high importance in the random forest and K-NN methods: 30% and 28%, respectively.

The importance of the amino acid sequences under different methods is shown in Figure 5B. Amino acid sequences provide information about the primary structure of proteins and are the basis for the formation of the secondary structure of proteins. Naive Bayes has the highest importance for amino acid sequences, which is about 25%, indicating that amino acid sequences provide independent and important information under the conditional independence assumption. The K-NN and random forest methods have the next highest importance for amino acid sequences: about 20% and 15%, respectively.

The importance of the trend factors under different methods is shown in Figure 5C. The trend factors reflect the conservatism and change trends of local regions in protein sequences, and these trends can reveal the formation patterns of secondary structures. The secondary structure trend factors (also known as amino acid compositional tendencies) proposed in this study reflect the intrinsic properties of amino acids and the compositional differences of different structures, which have significantly different effects on the secondary structure. In the α-helical structure, alanine and glutamic acid have a high preference due to the ability of their side chains to form hydrogen bonds and internal electrostatic interactions, whereas in the β-folded structure, glycine and alanine are more common because their side chains do not interfere with the formation of the β-sheet. Naive Bayes had the highest importance for the trend factor at about 25%, which may be due to the fact that the trend factor provides information that is independent of the other features and thus is particularly important in the NB model. K-NN also had a higher importance for the trend factor at about 21%, which reflects the role of the trend factor in the calculation of neighborhood distances.

The importance of the PSSM matrix under the different methods is shown in Figure 5D. The PSSM matrix provides evolutionary information in protein sequences, which reveals the conservation and variability at each position through multiple sequence comparison. This information plays an important role in capturing the stability and functionality of the protein secondary structure. SVM has the highest importance for the PSSM matrix, at about 48%, indicating that evolutionary information has a strong discriminatory power in high-dimensional feature mapping. The importance of random forests and K-NN to the PSSM matrix is about 40% and 31%, respectively.

The above analysis shows that the utilization of various factors varies significantly between different machine learning methods. Amino acid properties and trend factors play an important role in local information capture, while the evolutionary information provided by the PSSM matrix is prominent in support vector machines. The synergistic effect of these features helps to improve the accuracy and reliability of protein secondary structure prediction. These analyses lead to a better understanding of the properties as well as the role and performance of each factor.

### 3.5. Evaluation of the Effect of Different Combinations of Input Features

We also investigated the effects of different combinations of input features on protein secondary structure prediction. There are four main factors included in the multi-factor features, among which amino acid sequence information and the position specificity matrix are the more popular and major feature inputs nowadays, and there has been a lot of related research proving the contribution of these two factors to the field of protein secondary structure prediction; so, we take these two factors as the basic inputs. Meanwhile, amino acid properties and trend factors are used as additional ablation factors. The main core part of the multi-factor feature proposed in this study is the inclusion of the selected amino acid properties as well as the proposed trend factor; so, in this section, we collectively refer to the amino acid properties and the trend factor as the property feature (factor) to explore the effect of this property feature on the performance of protein secondary structure prediction.

**Figure 6:**
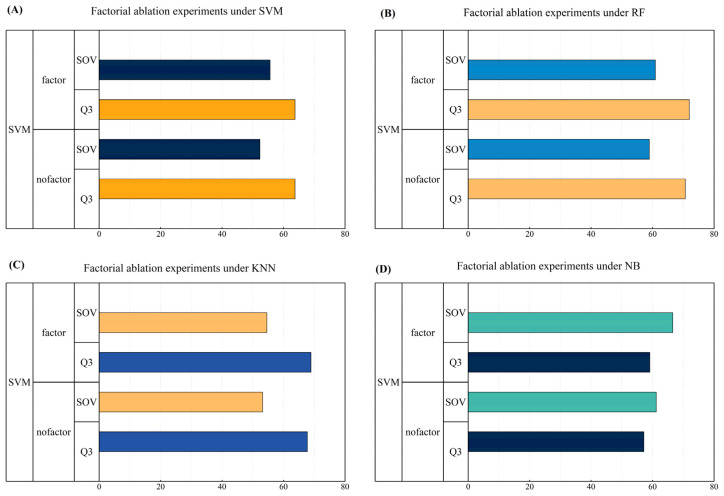
Demonstration of effect evaluation for different combinations of input features.

In Table 5, Table 6, Table 7 and Table 8, we show the performances of the four different machine learning methods with and without the inclusion of nature features, with overall visualization in Figure 6. The results show that the performance of the model with the inclusion of nature features is significantly higher than the performance of the model without nature features, by about two percentage points, regardless of which machine learning method is used. Therefore, we conclude that the nature features proposed in this study are important for multi-factor protein secondary structure prediction and significantly improve the prediction performance.

These findings highlight the importance of multiple factor features in protein secondary structure prediction and provide strong support for the further optimization of prediction models.

### 3.6. Case Study of Secondary Structure Prediction of Protein–Protein Interaction Complexes

In this study, we employed independent amino acid scoring as one of the methods for predicting the secondary structure of proteins. This method assesses the structural preference of amino acids by calculating the propensity score of each amino acid that is to be in a particular secondary structure (e.g., α-helix, β-fold, or randomly coiled). This scoring method is concise and intuitive, allowing a rapid assessment of each amino acid’s likelihood of being in a secondary structure. To apply these independent scores to the entire protein sequence, we calculated the total sequence score for the entire protein by accumulating the propensity scores for each amino acid. This method takes full account of the position of amino acids in the sequence and their influence on the overall structure and is thus able to reflect the tendency of the protein’s secondary structure distribution. Although the scores are based on independent scores for each amino acid, by accumulating these scores, we were able to obtain an overall understanding of the structural features of the entire protein sequence.

Building on this foundation, we extended this scoring method to apply it to the prediction of the secondary structure of complexes resulting from protein–protein interactions. Although our original method was designed to predict the secondary structure of a single protein, we can use this method to make secondary structure predictions for proteins in complex protein–protein interaction complexes. In extending the independent amino acid scoring method to apply it to these complexes, we first calculated the independent propensity scores for each amino acid in different secondary structures throughout the protein sequence. Although the score for each amino acid was calculated independently, we reasonably reflected the overall secondary structure characteristics of the protein by accumulating the scores for the entire protein sequence. Since the binding surface region usually plays a key role in interactions, the characteristics of the binding surface are reflected in the accumulated total score. Based on the local contribution assumption that the secondary structure of the binding surface is mainly dominated by the amino acid properties of these regions, we can categorize the binding surface region scores into sequence scores, and to a certain extent, it is possible to convert the complex data information into the independent amino acid scores used in this study; this binding surface information can be approximated as the additive effect of the individual amino acid scores, from which we obtain the total sequence scores for secondary structure prediction.

**Figure 7:**
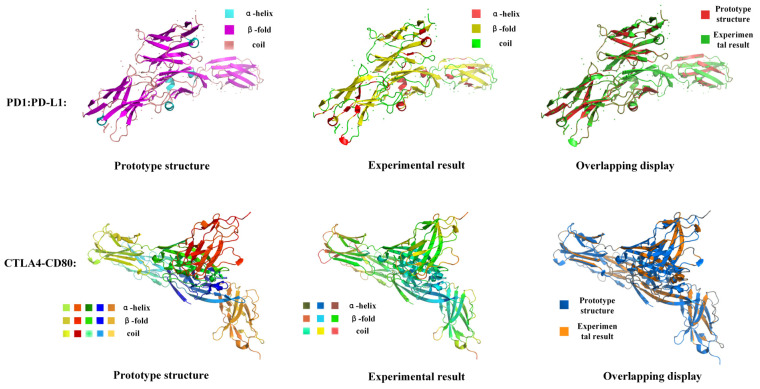
Display of PSSP prediction results for protein–protein interaction product complexes PD1:PD-L1 and CTLA4:CD80.

For this purpose, two widely studied protein–protein interaction complexes, PD1:PD-L1 and CTLA4:CD80, were selected for the case studies. By performing secondary structure prediction of these complexes, the practical application of the methodology of this study can be shown, as demonstrated in Figure 7.

In our presentation of the results, we observe a high overall level of agreement between the predicted structure and the prototype structure in terms of tertiary structure conformation. Overall, despite some secondary structural differences, the predicted results maintain a basic level of agreement with the prototype structure. In order to more visually demonstrate the prediction performance of our method, we performed an overlay display (overlapping display) of the prototype and predicted structures. This visualization method clearly demonstrates the ability of our model in capturing the tertiary structure of proteins and helps us to verify the accuracy and reliability of the predictions.

By demonstrating the secondary structure prediction results in tertiary structure conformations, we validate the applicability and effectiveness of the scoring method in dealing with more complex protein structures. These case studies not only demonstrate the application of our method in complex protein–protein interaction complexes, but also provide valuable practical references for future related studies.

## 4. Discussion and Conclusions

In this study, we quantitatively analyzed the importance of multi-factor features in protein secondary structure prediction and validated it with multiple sets of experiments using four classical and highly explanatory machine learning methods. The results show that multi-factor features play an important role in improving the accuracy of secondary structure prediction, in both three-state and eight-state prediction. Firstly, the position-specific scoring matrix (PSSM) performs well in multiple machine learning methods, especially SVM and RF. The PSSM factors contain rich protein evolution information, which reflects the probability of amino acid evolution at each position by quantifying the sequence comparison information. This makes PSSM very effective in capturing structure-related evolutionary information. Therefore, PSSM is more suitable for methods that can manage high-dimensional and intricate features, such as RNN and its variants LSTM and GRU. Such methods are equipped with the ability to handle high-dimensional data and complex features and are able to take full advantage of the rich information contained in PSSM through techniques such as feature extraction and feature processing. Secondly, amino acid sequences perform poorly in a variety of machine learning methods but perform relatively well in K-NN methods. Although amino acid sequences provide only first-level structural information, their existence is the indispensable foundation for all subsequent feature extraction. The relative dominance in K-NN suggests that amino acid sequence factors are more suitable for methods with linear processing capabilities and in pre- and post-correlation analyses. Typical of such methods is the Transformer model, which effectively captures global dependencies in sequences through a self-attentive mechanism, and despite the simplicity of the amino acid sequence information, its foundational status and linear correlation properties make this type of factor perform better with specific methods. Finally, the amino acid nature and trend factors did not perform well when analyzed individually, but in the combined analysis, these factors significantly improved the predictive performance. The combination of amino acid properties and trend factors can take advantage of local and action features to enhance the accuracy and stability of predictions. This combination of factors is suitable for models with stronger feature extraction capability, such as convolutional neural networks (CNNs), which can capture the local associations of amino acid properties and trend factors through convolutional operations and then give full play to the combination of factors. By reasonably combining these multi-factor features, the accuracy and stability of PSSP can be significantly improved. Finally, this study conducted a case study of the secondary junction prediction of protein–protein interaction complexes (e.g., PD1:PD-L1 and CTLA4:CD80) using an independent amino acid scoring method, and the results showed that the overall agreement between the predicted structures and the prototypical structures in the tertiary structural conformation was high, suggesting that the present method still has a better performance in more complex compound protein structures.

This research result provides a solid theoretical foundation for the further optimization of the models and the improvement of the prediction performance in the field of protein structure prediction in the future. Based on these findings, future researchers can optimize multi-factor feature combinations and apply them to deep learning models to enhance the prediction performance of the protein secondary structure. By taking full advantage of multi-factor features, researchers can further enhance the precision and dependability of prediction and promote the development and application of protein secondary structure prediction-related fields. These results not only provide an important reference for theoretical research, but also provide valuable experience for practical applications.

## Figures and Tables

**Table 1:** Property parameters of 20 amino acids.

**Amino Acid**	**R**	**K**	**H**	**P**	**A**	**L**	**I**	**V**	**W**	**G**
**H**	10.76	9.74	7.59	6.3	6	5.98	5.97	5.96	5.89	5.79
**pK_b2_** **(NH^3+^)**	2.17	2.18	1.82	1.99	2.34	2.36	2.36	2.32	2.83	2.34
**pl**	9.04	8.95	9.17	10.6	9.69	9.6	9.6	9.62	9.39	9.6
**pK_a1_** **(COOH)**	−2.53	−1.5	−0.4	0.12	0.62	1.06	1.38	1.08	0.81	0.48
**Amino acid**	**M**	**S**	**Y**	**Q**	**T**	**F**	**N**	**C**	**E**	**D**
**H**	5.74	5.68	5.66	5.65	5.6	5.48	5.41	5.07	4.25	3.65
**pK_b2_** **(NH^3+^)**	2.28	2.21	2.2	2.17	2.09	1.83	2.02	1.96	2.19	1.88
**pl**	9.21	9.15	9.11	9.13	9.1	9.13	8.8	10.28	9.67	9.6
**pK_a1_** **(COOH)**	0.64	−0.18	0.26	−0.85	−0.05	1.19	−0.78	0.29	−0.74	−0.9

**Table 2:** Hyperparameter optimization exploration of multiple machine learning methods.

Model	Data	Hyperparameter	H (Value)	Q3 (%)
Support Vector Machine	CB513	kernel function	Linear	56.03
			RBF	60.29
			Poly	58.74
			Sigmoid	54.00
Random Forest		decision tree	100	56.03
			110	57.51
			120	58.65
			130	57.01
K-Nearest Neighbor		K-value	1	52.00
			2	53.20
			3	54.79
			4	54.21

**Table 3:** Comparative analysis of the effectiveness of various machine learning methods for three-state secondary structure prediction.

Methods	CB513		CASP10		CASP11	
	$Q_{3}$ (%)	Sov (%)	$Q_{3}$ (%)	Sov (%)	$Q_{3}$ (%)	Sov (%)
SVM	60.29	56.25	63.73	55.61	64.49	51.16
Random Forest	58.65	56.25	71.92	60.88	63.67	57.64
K-Nearest neighbor	54.79	54.69	68.92	54.57	65.85	51.89
Naive Bayes	57.65	51.56	59.08	66.55	61.76	56.31

**Table 4:** Comparative assessment of the effectiveness of various machine learning methods for predicting eight-state secondary structure.

Methods	CB513		CASP10		CASP11	
	$Q_{8}$ (%)	Sov (%)	$Q_{8}$ (%)	Sov (%)	$Q_{8}$ (%)	Sov (%)
SVM	50.32	48.44	49.10	39.06	54.54	43.22
Random Forest	47.25	43.75	61.89	58.45	59.60	54.68
K-Nearest neighbor	42.65	34.38	59.33	61.57	56.92	48.32
Naive Bayes	48.34	42.68	47.12	51.26	50.16	48.37

**Table 5:** Study findings for varied sets of input features in support vector machines.

Data	Network	$Q_{3}$ (%)	Sov (%)
no factor	SVM	61.55	52.31
factor		63.73	55.61

**Table 6:** Study findings for varied sets of input features in random forest.

Data	Network	$Q_{3}$ (%)	Sov (%)
no factor	Random Forest	70.61	58.91
factor		71.92	60.88

**Table 7:** Study findings for varied sets of input features in K-nearest neighbor.

Data	Network	$Q_{3}$ (%)	Sov (%)
no factor	K-Nearest Neighbor	67.74	53.18
factor		68.92	54.57

**Table 8:** Study findings for varied sets of input features in naive Bayes.

Data	Network	$Q_{3}$	Sov (%)
no factor	Naive Bayes	57.15	61.17
factor		59.08	66.55

## Data Availability

The source code and dataset for this study have been uploaded to https://github.com/LindaEdu/Multi-Factor on 3 August 2024.
